# Heart failure and sudden cardiac death in heritable thoracic aortic disease caused by pathogenic variants in the *SMAD3* gene

**DOI:** 10.1002/mgg3.396

**Published:** 2018-05-01

**Authors:** Julie De Backer, Alan C. Braverman

**Affiliations:** ^1^ Department of Cardiology and Center for Medical Genetics Ghent University Hospital Ghent Belgium; ^2^ Cardiovascular Division Department of Medicine Washington University School of Medicine Saint Louis MO USA

**Keywords:** aortic aneurysm, thoracic, heart failure, *SMAD3*, sudden cardiac death

## Abstract

**Background:**

Predominant cardiovascular manifestations in the spectrum of Heritable Thoracic Aortic Disease include by default aortic root aneurysms‐ and dissections, which may be associated with aortic valve disease. Mitral‐ and tricuspid valve prolapse are other commonly recognized features. Myocardial disease, characterized by heart failure and/or malignant arrhythmias has been reported in humans and in animal models harboring pathogenic variants in the *Fibrillin1* gene.

**Methods:**

Description of clinical history of three cases from one family in Ghent (Belgium) and one family in St. Louis (US).

**Results:**

We report on three cases from two families presenting end‐stage heart failure (in two) and lethal arrhythmias associated with moderate left ventricular dilatation (in one). All three cases harbor a pathogenic variant in the *SMAD3* gene, known to cause aneurysm osteoarthritis syndrome, Loeys‐Dietz syndrome type 3 or isolated Heritable Thoracic Aortic Disease.

**Conclusions:**

These unusual presentations warrant awareness for myocardial disease in patients harboring pathogenic variants in genes causing Heritable Thoracic Aortic Disease and indicate the need for prospective studies in larger cohorts.

## 1 INTRODUCTION

In recent years, both the genetic and clinical spectrum of Heritable Thoracic Aortic Disease (H‐TAD) has expanded substantially. H‐TAD comprises a heterogeneous group of disorders with as a common denominator aortic aneurysm or dissection on one or several levels of the aorta (Pyeritz, 2014).

Currently, known causal genes that have been identified in H‐TAD are commonly grouped into those affecting structure (i.e., genes encoding extracellular matrix (ECM) components (*FBN1*(*134797), *COL3A1* (*120180), *MFAP5*(*601103), *ELN* (*130160), *EFEMP2* (*604633)) and those that affect the ability to modify structure in response to changes in mechanical load imposed on the aortic wall. The latter group can be divided into genes encoding various proteins involved in TGFβ signaling (TGFBR15*190181), *TGFBR2* (*190182), *TGFB2* (*190220), *TGFB3* (*190230), *SMAD3* (*603109) and genes encoding proteins involved in vascular smooth muscle cell contractility (*ACTA2* (*102620), *MYH11* (*160745), *MYLK* (*600922), *PRKG1* (*176894), *FLNA* (*300017)).

From a clinical cardiovascular perspective, the spectrum broadened as well with attention also being paid to extra‐aortic cardiovascular manifestations. There has been growing evidence of intrinsic myocardial dysfunction in patients with Marfan syndrome (MFS) caused by pathogenic variants in the *FBN1* gene (Alpendurada et al., 2010; de Backer et al., 2006; Kiotsekoglou et al., 2008; Loeper et al., 2016; Rybczynski et al., 2007). Clinically overt heart failure, even necessitating cardiac transplantation has been reported in some cases and small series (Audenaert, de Pauw, François, & De Backer, 2015; Kesler et al., 1994; Knosalla et al., 2007) but the majority of patients will present subclinical forms of myocardial dysfunction, the clinical impact of which is not entirely understood yet. It cannot be excluded that myocardial involvement conveys an increased risk for arrhythmias. Indeed, there are several reports of an increased risk of ventricular arrhythmia and SCD, related or not related to underlying myocardial dysfunction (Chen, Fagan, Nouri, & Donahoe, 1985; Hoffmann et al., 2012; Schaeffer et al., 2015; Yetman, Bornemeier, & McCrindle, 2003). A correlation between the risk for ventricular arrhythmias and ejection fraction and NTpro‐BNP levels has been documented in several studies of these studies. Notably, heart failure and sudden cardiac death in MFS has also been reported as a major cause of death in earlier series and surgical reports (Gott et al., 1999; Murdoch, Walker, Halpern, Kuzma, & McKusick, 1972).

One small study investigated the genotype in relation to left ventricular (LV) function and noticed that LV dilatation in MFS patients is more often seen in patients with a non‐missense *FBN1* pathogenic variant and in those patients without an *FBN1* pathogenic variant (Aalberts et al., 2014). Whether this latter group included patients harboring variants in other H‐TAD genes is not known.

More recently, the observation of myocardial dysfunction was also confirmed in various mouse models for MFS (Campens et al., 2015; Cook et al., 2014; Rouf et al., 2017; Tae, Petrashevskaya, Marshall, Krawczyk, & Talan, 2016). The exact mechanism of these findings is largely unexplained. Animal experimental studies may indicate a perturbation in TGFβ signaling or a malfunction in mechanobiology, especially in mechanical sensing (Cook et al., 2014). The importance of the TGFβ pathway in the aortic pathology of various HTAD entities has already been demonstrated. Whether patients with pathogenic variants in these genes also have an increased risk of developing cardiomyopathy or arrhythmias is less clear.

*SMAD3* pathogenic variants can give rise to a form of H‐TAD, also characterized by osteoarthritis, hence the designation Aneurysm Osteoarthritis Syndrome (van de Laar et al., 2011). Some patients with *SMAD3* pathogenic variants exhibit typical characteristics of Loeys‐Dietz syndrome (LDS), from which the term LDS type 3 is sometimes used while others present very little outward syndromic features and the term “Familial or Heritable Thoracic Aortic Disease” may be more appropriate (Regalado et al., 2011).

In a first published retrospective series on the clinical cardiovascular characteristics of Aneurysm Osteoarthritis patients, left ventricular hypertrophy was established in 19% of a cohort of 44 patients (van der Linde et al., 2012). Patients also are reported to have a higher BNP values and an increased PVC burden (van der Linde et al., 2012). Of the 15 registered deaths in this series, six were due to aortic dissection, two from heart failure and three related to sudden cardiac death.

Herein, we report two families (three cases) with a pathogenic *SMAD3* variant displaying severe heart failure (two cases) and sudden cardiac death (one case), indicating a possible relationship between *SMAD3* pathogenic variants and these manifestations to heighten awareness of this association and to further evaluate in larger series.

## 2 ETHICAL COMPLIANCE

The patient (in the St Louis case) and family (in the Ghent case) gave written consent for publication.

## 3 CASES

### 3.1 St Louis

At the age of 40 years old, the proband was referred for further evaluation in the setting of familial occurrence of aortic aneurysms and dissection. His medical history was notable for bilateral inguinal hernia correction. Aside from a mild pectus excavatum and an increased arm span (ratio 1.06), he did not present any outward features reminiscent of MFS or LDS. Echocardiography revealed an aortic root aneurysm of 50 mm. Family history revealed a presumed diagnosis of “Marfan syndrome” in his deceased father and several other family members on his father's side known with aortic aneurysms and dissections. No one was known to have lens dislocation. Preoperative evaluation demonstrated left ventricular dysfunction (LVEF 35%) and normal coronary arteries. Valvular heart disease was excluded on cardiac ultrasound. The patient underwent valve‐sparing aortic root replacement. Despite therapy with ACE inhibitors or angiotensin receptor blockers and beta blocker, he developed progressive and severe heart failure (LVEF 15%). An implantable cardiac defibrillator (ICD) was placed for primary prevention of sudden cardiac death. Brief episodes of nonsustained ventricular tachycardia were noted on subsequent ICD recordings. In the year following diagnosis, heart failure worsened despite inotropic support, and a HeartMate II^®^ left ventricular assist device was inserted as a bridge to transplant. Genetic testing demonstrated a pathogenic *SMAD3* variant (c.859 C>T; p.Arg287Trp; NM_005902.3). This variant has been reported previously by van de Laar and colleagues (van de Laar et al., 2011) in a large family with several patients presenting with Aneurysm Osteoarthritis syndrome. Heart failure is not mentioned but interestingly, two family members are reported with sudden cardiac death at a young age (35 and 39 years; Table 1).

**Table 1:** Summary of clinical and genetic findings in the three reported cases

		Cardiovascular features	Other clinical manifestations	Family history	Genetic findings
St Luis case		Aortic Root Aneurysm (50 mm @ age 40 years)—LVEF 35% R/Valve Sparing ARR Postop LVEF ↓ 15%—R/LVAD NSVT—R/ICD	Inguinal hernia Mild Pectus deformity Armspan/length ratio 1.06	Father died of “Marfan syndrome” Several other paternal family members presenting aortic aneurysm/dissection	*SMAD3* c.859 C>T; p.Arg287Trp
Ghent case	Father	“Idiopathic Dilated Cardiomyopathy” @ age 25 years Aortic dissection upon LVAD insertion—died @ age 27 years	Long fingers	Mother and maternal aunt aortic dissection in 3rd decade of life	*SMAD3* c.584‐585inTC; p.Gln195HisfsX3
	Daughter	Mitral valve prolapse Ventricular Extrasystole's LV dilatation (LVEDD 61 mm @ 21 years) Normal aortic root dimensions Out of Hospital Arrest—died @ age 22 years	Arachnodactylia Flat feet Joint laxity Retropatellar cartilage problems		

ICD, implantable cardiac defibrillator; LV, left ventricle; LVEDD, left ventricular end diastolic diameter; LVEF, left ventricular ejection fraction; LVAD, left ventricular assist device; NSVT, nonsustained ventricular tachycardia.

### 3.2 Ghent

The proband in this family is a young girl, presenting for the first time at the age of 5 years after the death of her father. Her father had been diagnosed with idiopathic dilated cardiomyopathy at the age of 25 years. Valvular heart disease was excluded. He developed intractable heart failure at the age of 27 and he was listed for heart transplantation. He presented with acute aortic dissection after insertion of a LVAD and died. Both his mother and maternal aunt had died in their 3rd to 4th decade of life from aortic dissection. At the age of 5 years, the proband presented some mild Marfanoid features with joint laxity, flat feet, arachnodactylia slender build, mild myopia and mitral valve prolapse. Genetic screening of the *FBN1* gene revealed the presence of a benign polymorphism. Over the years, follow‐up showed mild scoliosis occurring in adolescence and stretch marks. Myopia was not progressive. She developed retropatellar cartilage problems in both knees. She was treated throughout childhood with verapamil for symptomatic ventricular extrasystoles. Aortic dimensions at all levels have always been normal. At the age of 21 years, moderate dilatation of the left ventricle was noted on echocardiography (left ventricular end diastolic diameter 61 mm) with preserved systolic and diastolic function. Aortic root diameter (sinus of Valsalva) was 32 mm—*z*‐score 0.24). Holter monitoring revealed multiple ventricular couplets (5% ventricular ectopy, 15 couplets). An electrophysiologic study did not demonstrate any inducible arrhythmias. At the age of 22 years, she presented with an out‐of‐hospital cardiac arrest. She was resuscitated by her mother and ventricular fibrillation was documented by the EMS. Severe brain damage ensued and she died 10 days later. An autopsy excluded aortic dissection, coronary artery anomalies or cerebral vascular abnormalities. A new genetic screening using a NGS panel including *FBN1, TGFRB1/2, ACTA2,* and *SMAD3* revealed a pathogenic variant in the *SMAD3* gene (c.584_585insTC; p.Gln195Hisfs*3). This variant has not been reported elsewhere. Furthermore cascade screening in the family revealed its presence in one man presenting severe mitral valve regurgitation due to prolapse, necessitating valve replacement. His 40‐year‐old son also harbors the variant but has no cardiovascular manifestations so far.

## 4 DISCUSSION

The notion of increased rates of heart failure/myocardial dysfunction and ventricular arrhythmias/sudden cardiac death in patients with Marfan syndrome (MFS) has been established for several decades, both in mortality registries (Gott et al., 1999; Murdoch et al., 1972) as in clinical studies (Alpendurada et al., 2010; Campens et al., 2015; Cook et al., 2014; Hoffmann et al., 2012; Kiotsekoglou et al., 2008, 2009; Loeper et al., 2016; Rouf et al., 2017; Rybczynski et al., 2007; Tae et al., 2016). The exact nature of these abnormalities is largely unknown. Suggested triggers/modifiers for myocardial dysfunction and arrhythmias include volume overload induced by valvular regurgitation or pressure overload by aortic arch constriction (in animals). Several studies have, however, indicated that myocardial dysfunction may occur in the absence of any of these triggers, suggesting an intrinsic myocardial defect (Alpendurada et al., 2010; de Backer et al., 2006). Some studies in animal models indicate a possible role for altered TGFβ signaling. Given the known phenotypic overlap between MFS and other HTAD entities related to genes encoding components of the TGFβ signaling pathway, myocardial dysfunction and arrhythmias may also be present. Limited data on patients with *SMAD3* pathogenic variants are available at present with reports of sudden cardiac death and left ventricular hypertrophy in one of the initial series on Aneurysm Osteoarthritis Syndrome due to *SMAD3* pathogenic variants (van der Linde et al., 2012).

Here, we report the clinical summaries of three patients harboring a pathogenic *SMAD3* variant and displaying idiopathic dilated cardiomyopathy requiring advanced mechanical support in two cases and left ventricular dilatation complicated by malignant arrhythmias in one case.

To further investigate the relevance and underlying mechanisms of these findings, larger scale (cohort and prospective) studies are needed. Meanwhile awareness for the problem and appropriate investigation and management in patients with suggestive symptoms is warranted.

## CONFLICT OF INTEREST

The authors have no conflict of interest with regard to this research.
